# The Use of Telepsychiatry Services in Emergency Settings: Scoping Review

**DOI:** 10.2196/51814

**Published:** 2024-07-15

**Authors:** Ligat Shalev, Renana Eitan, Adam J Rose

**Affiliations:** 1 School of Public Health, Hebrew University Jerusalem Israel; 2 Psychiatric Division, Sourasky Medical Center Tel Aviv-Yafo Israel

**Keywords:** implementation science, emergency department, telepsychiatry, organizational innovation, eHealth, mHealth, scoping review, implementation, psychiatric patient, clinical outcome, rural, feasible, acceptable, effectiveness, electronic database

## Abstract

**Background:**

Telepsychiatry (TP), a live video meeting, has been implemented in many contexts and settings. It has a distinct advantage in the psychiatric emergency department (ED) setting, as it expedites expert assessments for psychiatric patients. However, limited knowledge exits for TP’s effectiveness in the ED setting, as well as the process of implementing TP in this setting.

**Objective:**

This scoping review aimed to review the existing evidence for the administrative and clinical outcomes for TP in the ED setting and to identify the barriers and facilitators to implementing TP in this setting.

**Methods:**

The scoping review was conducted according to the guidelines for the PRISMA-ScR (Preferred Reporting Items for Systematic Reviews and Meta-Analyses extension for Scoping Reviews). Three electronic databases were examined: PubMed, Embase, and Web of Science. The databases were searched from January 2013 to April 2023 for papers and their bibliography. A total of 2816 potentially relevant papers were retrieved from the initial search. Studies were screened and selected independently by 2 authors.

**Results:**

A total of 11 articles were included. Ten papers reported on administrative and clinical outcomes of TP use in the ED setting and 1 on the barriers and facilitators of its implementation. TP is used in urban and rural areas and for settings with and with no on-site psychiatric services. Evidence shows that TP reduced waiting time for psychiatric evaluation, but in some studies, it was associated with prolonged total length of stay in the ED compared with in-person evaluation. Findings indicate lower admission rates in patients assessed with TP in the ED. Limited data were reported for TP costs, its use for involuntary commitment evaluations, and its use for particular subgroups of patients (eg, those with a particular diagnosis). A single paper examined TP implementation process in the ED, which explored the barriers and facilitators for implementation among patients and staff in a rural setting.

**Conclusions:**

Based on the extant studies, TP seems to be generally feasible and acceptable to key stakeholders. However, this review detected a gap in the literature regarding TP’s effectiveness and implementation process in the ED setting. Specific attention should be paid to the examination of this service for specific groups of patients, as well as its use to enable assessments for possible involuntary commitment.

## Introduction

### Telepsychiatry Use Over the Years

The history of telepsychiatry (TP) began with doubts about its use [1,2]. While there are still questions about TP, it has gained increasing acceptance in recent years, as reflected through changes in relevant regulations [3]. TP is used for psychiatric assessment, treatment, and follow-up [4]. So far, the most prevalent technologies used for TP are by telephone [5], email [6], or recorded or live videos and hybrid models [7]. TP is used in the private [8] and public sectors, including for primary care [9] and secondary care [10]. TP has also been delivered in clinical settings [10] and in patients’ home environments [11]. TP has been used to treat different mental health conditions, and in different situations, including suicide attempts, self-harm, schizophrenia, and dual diagnosis of mental health conditions with substance abuse [12]. TP has been adapted to different treatment approaches, including for individuals [13] and group sessions [14]. TP has been used in both urban [15] and rural areas [11].

### Current Evidence for Effectiveness of TP and Regarding Its Implementation

Various studies have examined the effectiveness of TP, often compared with face-to-face treatment approaches. In terms of accuracy of diagnosis and treatment decisions, TP has been shown to be as accurate as meeting with patients in person [16,17]. Using TP has been shown to reduce emergency department (ED) length of stay (LOS) by allowing more rapid access to psychiatric expertise [17]. For similar reasons, TP has been shown to reduce admission rates [18]. TP has been used to provide on-site psychiatric services to hospitals that previously did not have any [19]. Both patients [20] and providers [17] showed high satisfaction rates.

TP has also been examined using cost-effectiveness analyses [21], and at least some studies have found that it is cost saving compared with usual care [22,23]. Other studies have examined the process of implementing TP in different settings [9,24-26]. For example, some studies have detailed the experience of implementing remote mental health consultations during the COVID-19 [27], or reasons why some ED directors are avoiding the use of remote services, including TP [28].

### Specific Challenges When Using TP for Psychiatric Emergencies

TP has advantages for general use, but it may have a particularly important role in addressing psychiatric emergencies. Most people find the ED an uncomfortable place to be [29]. However, for patients experiencing psychiatric emergencies, the ED may be even worse. The ED may exacerbate patients’ agitation, which may put health care providers or other bystanders at risk for violence [29]. In addition to this immediate effect, the ED can also have a long-term effect on psychiatric patients. Faessler and colleagues [30] found that psychological distress could last up to 30 days after ED discharge for patients with psychiatric disorders. Considering these data, TP may be a highly useful solution for ED settings, if it can help minimize patients’ time in the ED [31].

In the last few years, several reviews summarized the current evidence of TP services in the ED setting. One study reviewed the current data on acute situations but included not just psychiatric services but other practices and also included home-based services in addition to the ED setting [11]. A second review examined the barriers and facilitators of implementing TP, but most of the studies that were included did not focus on the use of TP for emergency settings [26]. A third study reviewed TP interventions in emergency and crisis situations, but this review included studies published more than a decade ago, when video-link technology was much less developed [32]. Thus, no updated published review of TP use for adult emergencies is available.

### Objectives

TP may bridge critical gaps in mental health care access and quality, addressing key issues, such as prolonged waiting times or workforce shortages, that hinder timely and effective patient care. Thus, TP has considerable potential to help improve service delivery and outcomes in mental health. To our knowledge, reviews assessing the use of TP in emergency settings in the past 10 years have not been conducted. Given the limited evidence, the objectives of this review were to search the literature on psychiatric live video meeting in emergency settings, to assess the overall findings regarding clinical and implementation outcomes, and to enumerate the barriers and facilitators for successful implementation.

## Methods

### Overview

We conducted this scoping review following the methodological guidance proposed by Arksey and O’Malley [33], Levac et al [34], and The Joanna Briggs Institute Reviewers’ Manual [35]. The 5 stages used in this scoping review were based on guidelines from Arksey and O’Malley: (1) identifying the research question; (2) identifying the relevant studies; (3) study selection; (4) charting the data; and (5) collating, summarizing, and reporting the results [33]. Our study focused on the current administrative and clinical evidence regarding the use of TP services in the ED setting, as well as the factors affecting their implementation in the ED setting. The reporting of this scoping review was guided by the PRISMA (Preferred Reporting Items for Systematic Reviews and Meta-Analyses) extension for Scoping Reviews (PRISMA-ScR) checklist [36] (Multimedia Appendix 1).

### Search Strategy

We searched 3 electronic databases, including PubMed, Embase, and Web of Science, using the following terms and combinations: (1) psychiatry, mental health, mental disorder, mental health care, and mental disease; (2) telepsychiatry, telemedicine, virtual medicine, tele health, eHealth, telecare, tele emergency, and digital mental health; and (3) video, video conference, videoconferencing, conference meeting, streaming, zoom, remote consultation, long distance consultation, distance counseling, eCounseling, and web-based counseling (Multimedia Appendix 2).

### Inclusion and Exclusion Criteria

Studies were included based on the inclusion and exclusion criteria, and if they met the population, concept, and context categorization recommended by The Joanna Briggs Institute [35] (Table 1). We included studies focused on individuals 18 years of age and older who had a psychiatric session. Due to the different nature of the following patient groups, they were excluded from the search: children, couple or group sessions, and patients who have been arrested or convicted. While it was allowable for the clinician to address substance abuse as part of the service, we did not include studies solely addressing substance abuse issues. We also focused solely on 2-way video assessment and excluded other modes of communication such as telephone or asynchronous text messages.

We did make an exception to our rule about patient ages to include 3 especially important and broad-based studies. These studies were conducted nationwide [37,38] and statewide [15] and involved patients of all ages. We also focused on studies conducted within the past 10 years, due to significant advances in video-link technology around that time. We focused on the dates of data collection rather than publication, since a variable period may elapse between data collection and publication. Two studies started data collection on October 2012 for and finished collecting data years later; we decided to include these studies [39,40].

**Table 1:** Inclusion and exclusion criteria.

Inclusion criteria		Exclusion criteria
**Participants**		
	Patients who are 18 years of age and older or ED^a^ staff that use and report about the telepsychiatry service based on direct experience	Children and teenagers younger than 18 years
	Individuals	Couple, family, or group Forensic cases, correctional facilities, or services focused primarily on delivering treatment for substance abuse Not clinician-to-clinician telepsychiatry service (advice of psychiatrists to general clinicians about patients)
**Concept**		
	Psychiatry services	Other services than psychiatry (eg, psychology, neurology, or social work)
	Use of live video communication	Asynchrony video communication, telephone, text chat, email, app, or video game
**Context**		
	Emergency settings	Nonemergency settings or acute services in the community setting
**Type of study**		
	Qualitative, quantitative, or mixed methods studies. Quantitative studies must describe results of at least 30 participants	—^b^
	Empirical data with detailed methodology presented in journals, editorials, commentaries, letters to the editor, or scientific reports	Nonempirical data or empirical data with insufficient description of study methodology. Also, conference abstracts, essays, book chapters, and books, and development of research tools without pilot-testing
	Studies that use one of the following designs: observational and experimental, cross-sectional, or longitudinal; randomized controlled trials, nonrandomized or noncontrolled trials	Case series or case studies
	Data collected during the past 10 years (from 2013)	—
**Language**		
	English	Languages other than English

^a^ED: emergency department.

^b^Not applicable.

### Screening and Selection of Studies

**Figure 1:**
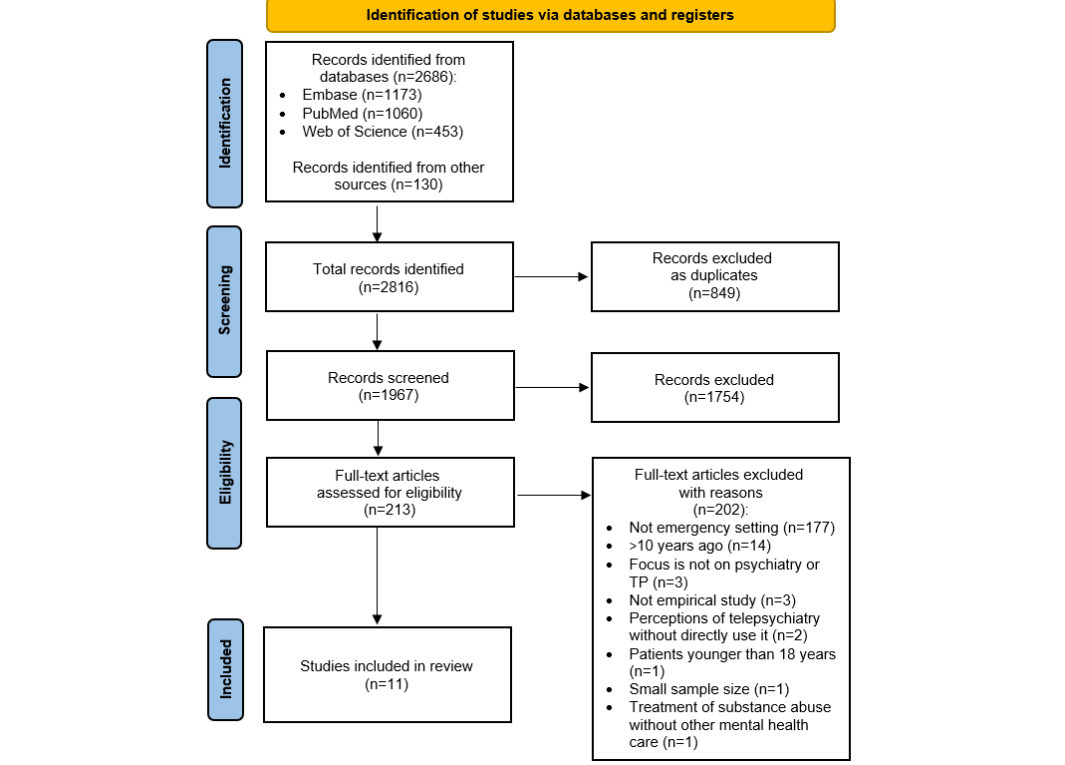
PRISMA (Preferred Reporting Items for Systematic Reviews and Meta-Analyses) flowchart. TP: telepsychiatry.

The initial search of the 3 databases yielded 2,686 results. The hand search of the selected papers’ bibliography identified 130 additional records. After duplicates were removed, 1967 (69.8%) records were reviewed. The titles and abstracts of 69.8% (1967/2816) of the articles were screened, and 89.2% (1754/1967) of the articles were excluded as being not relevant. LS performed the initial screening to identify articles that were clearly not relevant, keeping articles that were questionably relevant or probably relevant. Then LS and AR performed independent full-text review of the 213 retained articles. A total of 94.8% (202/213) articles were excluded based on the reasons shown in Figure 1. A total of 11 publications were ultimately included in the scoping review. In case of disagreement, LS and AR discussed the article until agreement was reached. The reasons for exclusion, as well as the entire selection procedure, are shown in the PRISMA flow diagram (Figure 1).

### Charting the Data

The articles included in this scoping review were reviewed and results were recorded using a Microsoft Excel (Microsoft Corp) data charting table. The table included general information about the study characteristics (authors, publication year, title, data collection period, country, study purpose, study design, setting, and sample), a description of the TP service and usual care if applicable, outcome measures (administrative and clinical or process outcomes), and main study results.

### Collating, Summarizing, and Reporting the Results

Included studies were examined thoroughly to understand similarities and differences. We had the following main categories of study outcomes: (1) *administrative outcomes* (eg, ED LOS, or mental illness spending); (2) *clinical outcomes* (eg, admission disposition or patient and providers satisfaction); and (3) *process outcomes* of TP implementation.

## Results

A total of 11 articles were included for data extraction in this scoping review, as can be seen in the PRISMA flowchart (Figure 1).

### Characteristics of the Studies

Although the search strategy was from January 2013 to April 2023, the studies that were identified and included collected data between October 2012 and 2023, and were published between 2015 and 2023. Of the 11 articles, 9 were published in the United States [15,19,37-43],1 in Australia [44], and 1 in Israel [45]. Of the 11 studies, 10 [15,19,37-43,45] were quantitative studies that examined administrative or clinical measurements of TP use, and 1 was a qualitative study that examined process outcomes [44]. Of the 10 quantitative studies, 3 were non–randomized controlled studies [40,41,45], 3 cross-sectional studies [15,19,38], 3 cohort studies [37,39,43], and 1 randomized controlled study [42] (Table 2).

In the next section, we will summarize the results of our review, organized by our 2 main research questions, namely (1) to review the existing evidence for both administrative and clinical outcomes of TP in the ED setting and (2) to identify the known barriers and facilitators to implementing TP in this setting.

First research question: what is the existing evidence for both administrative and clinical outcomes of TP in the ED setting?

**Table 2:** Characteristics of studies included in the scoping review.

Authors (years)	Countries	Study objective	Setting	Study sample	Methods (study paradigm, design, and tools)	Main outcomes measures and special characteristics
Freeman et al (2023) [37]	United States	To investigate ED^a^ TP^b^ usage in the pre–COVID-19 era	EDs Nationwide Rural and urban	69 EDs using TP from surveys conducted in 2017 and 2019	Quantitative Cohort study Questionnaire	Characteristics of EDs using TP ED LOS^c^ Patient disposition
Bistre et al (2022) [45]	Israel	To evaluate the reliability and acceptability of TP assessments compared with in-person for involuntary admissions	Psychiatric ED Regional Urban	38 patients were evaluated both by TP and in person	Quantitative Non-RCT^d^ study Interrater agreement	TP reliability Psychiatrists’ certainty in TP evaluations, including need for admission Psychiatrists’ and patients’ satisfaction of TP
Patel et al (2022) [19]	United States	To assess the impact of a TP service compared with in-person in EDs on admissions, mental illness spending, ED LOS, mental illness outpatient follow-up care, and mortality	EDs across 22 states Nationwide Urban and rural	134 EDs using TP 134 EDs not using TP	Quantitative Cross-sectional study Medical records	Characteristics of EDs with and with no TP ED LOS for psychiatric patients Patient disposition Mental health spending
Saeed et al (2022) [39]	United States	To study the impact of a TP service in reducing hospitalizations and cost savings	Hospital EDs Statewide (North Carolina) Remote	30 EDs using TP	Quantitative Cohort study Medical records	Involuntary admissions Mental health spending
Zhong et al (2021) [15]	United States	To examine the impact of a TP service compared with in-person across EDs on visit dispositions	EDs Statewide (New York) Urban and rural	18 EDs using TP 115 EDs not using TP	Quantitative Cross-sectional study Questionnaire and medical records	Characteristics of EDs with and with no TP Patient disposition
Brenner et al (2020) [41]	United States	To assess turnaround time with and with no TP and patients’ satisfaction of TP	Three general hospital EDs Regional Urban	206 TP visits 186 in-person visits	Quantitative Non-RCT study Medical records and questionnaire	ED waiting time for psychiatric evaluation Patient satisfaction
Freeman et al (2020) [38]	United States	To investigate the prevalence of TP use for mental health in general EDs	EDs Nationwide Rural and urban	885 EDs using TP 3525 EDs not using TP	Quantitative Cross-sectional study Questionnaire and medical records	Characteristics of EDs with and with no TP ED LOS Patient disposition
Kothadia et al (2020) [40]	United States	To examine differences in patient disposition for ED psychiatric patients with and with no TP service	Hospital EDs Statewide (North Carolina) Remote	30 EDs with active and inactive periods of TP use 44,857 TP visits 42,074 in-person visits	Quantitative Non-RCT study Medical records	Patient disposition
Roberge et al (2020) [42]	United States	To assess whether TP use for mental health in the ED decreases hospitalization	Six EDs Regional Urban	323 TP visits 314 in-person visits Randomized	Quantitative RCT study Medical records and questionnaires	Patient disposition Suicide and self-harm diagnosis
Fairchild et al (2019) [43]	United States	To determine the effects of a TP service on clinical, temporal, and cost outcomes for patients	Four hospital EDs Regional Disadvantaged counties Rural	287 TP visits 153 in-person visits	Quantitative Cohort study Medical records	ED LOS and waiting time for psychiatric evaluation Patient disposition Suicide and self-harm diagnosis Mental illness spending
Saurman et al (2015) [44]	Australia	To examine the experience of implementing and using TP for mental health in an ED setting,	Seven EDs Regional Rural and remote	12 ED providers	Qualitative Interviews	Process outcomes of TP implementation

^a^ED: emergency department.

^b^TP: telepsychiatry.

^c^LOS: length of stay.

^d^RCT: randomized controlled trial.

### Scope and Location of Studies

The 10 studies that examined administrative and clinical outcomes in EDs varied in terms of the research population, study scope, and location. Three of the studies were conducted nationwide in the United States [19,37,38], 3 statewide (2 in North Carolina and 1 in New York) [15,39,40], and 4 regional in 1 or several local EDs [41-43,45]. Of the 10 studies, 3 provided TP services only in rural or remote areas [39,40,43], 3 in urban areas [41,42,45], and 4 in a mix of urban and rural areas [15,19,37,38]. Of the 4 studies with rural and urban EDs, 2 reported that most of the TP use occurred in urban areas [15,19] and 2 in rural areas [37,38]. It is worth noting that none of the included studies focused primarily on a comparison between the use of TP in rural and urban areas.

### On-site Psychiatric Services and TP Services

Some papers reported whether the medical centers using TP in fact had on-site psychiatric services some of the time, as opposed to having none at all. Three studies reported that less than 20% of their study sites lacked on-site psychiatric service [15,37,38], while 1 study reported that 65% of their study sites lacked on-site psychiatric service [19], and only 1 study reported that all EDs included in their sample lacked on-site psychiatric services [41]. In addition, TP was reported to be the only form of emergency psychiatric services for more than half of the EDs that participated in 2 nationwide studies in the United States in 2017 and 2019 [37,38].

### Effect of TP on Waiting Times in EDs

Two studies examined the effect of TP on waiting time from ED arrival until psychiatric assessment [41,43], while 4 examined the impact on ED LOS from arrival to discharge or admission [19,37,38,43]. Both studies that examined waiting time for psychiatric evaluation found it significantly lower for TP evaluation compared with in-person [41,43]. Of the 4 studies that examined total ED LOS, 2 showed a significant prolonged ED LOS for TP compared with in-person visits [19,43]; the other 2 studies showed similar prevalence in ED LOS for the same EDs in 2017 and in 2019 [37,38].

### Effect of TP on Discharge, Admission, and Transfer to Another Facility

Six studies examined the impact of TP on discharge, admission, and transfer to another facility. Three studies found that TP was associated with significantly lower admission rates compared with in-person visits [19,40,43];1 study showed no significant differences in admission rates between TP and in-person evaluation (55% vs 63%; *P*=.06) [42]; and 1 study found that EDs that used TP had significantly more admissions than EDs without this service (14% vs 12%; *P<*.001) [15]. One study examined whether TP had an impact on the rate of transfers to another facility. The findings were nuanced; total rates of transfer were lower, but among patients with a LOS of 1-2 days the rate of transfer was higher than with in-person care [40]. Another study that examined patients’ transfer to another facility did not find significant differences between TP patients (31%) compared with in-person (24%) [43].

### TP Costs

Several studies reported on the costs involved with TP. Saeed and colleagues [39] had examined the cost impact of 19,383 TP visits to 30 EDs in North Carolina. Seventy percent of the visits were encounters for involuntary commitments, and of these, 34% were converted to voluntary hospitalizations sometime before the end of the hospital stay, through a TP encounter. The aggregate cost savings for these conversions of involuntary to voluntary hospitalizations were more than US $20 million [39].

A national study in 22 US EDs found a significant increase in admissions for TP visits compared with in-person visits, which resulted with a significant increase in spending in a 90-day follow-up analysis [19]. Another study examined 3 diagnosis groups and found that the significantly most expensive TP visits were for substance abuse cases (US $4556), followed by suicide and self-harm cases (US $3559), and anxiety, mood, and other health disorders case (US $3355) [43].

### Data on Involuntary Commitment Cases

We found limited evidence regarding evaluations for involuntary commitment via TP. As mentioned earlier, Saeed et al [39] examined cost impact of using TP to enable staff to convert involuntary commitments into voluntary hospitalizations. In another study focused on examining the accuracy of TP compared with in-person evaluations, Bistre et al [45] evaluated the reliability of TP assessments compared with in-person assessments for involuntary admissions. An interrater agreement on recommended disposition and on indication for involuntary admission between raters was high [45]. Psychiatrists’ perceived certainty rates were high for both TP and in-person evaluations. Participants reported a high level of satisfaction with both TP and in-person evaluations, which were not significantly different [45]. In a separate study, patients reported that they were highly satisfied with TP use in the ED, although it was not used to evaluate for involuntary commitment [41].

### Special Behavioral Diagnostic Groups

Some studies have at least implied that TP may not be suitable for some groups of patients that require special attention. A study conducted in the United States found that TP was associated with a reduced wait time until psychiatric assessment, but a longer total ED LOS, compared with usual care. Interestingly, 36% (102/287) of the participants in the TP group were diagnosed as suicide and self-harm, compared with 22% (34/153) in the control group. This study also reported that the time from the end of TP assessment to disposition or discharge was significantly longer for patients with suicide and self-harm than for patients who were diagnosed with anxiety, mood, and other mental health disorders [43]. Those findings are implying that the poor TP performance may be related to the enlarged diagnosis group that requires more observation in the ED and not a result of TP use. In a national study that included patients with different diagnosis, TP was associated with longer ED LOS, more admissions, and greater costs. Yet, a nonsignificant higher rate of suicide and self-harm cases was found in the TP group (4925/35,861, 14%) compared with the in-person group (3734/34,982, 11%), suggesting, again, that the results may be affected by differences in patient characteristics between the TP group and the control group [19].

Having examined the existing evidence for TP’s impact on administrative and clinical outcomes in EDs, we will now move to the second research question. In the following section, we will describe the current evidence about the barriers and facilitators to implementing TP in these settings, understanding that successful implementation hinges on navigating these elements.

Second research question: what are the known barriers and facilitators to implementing TP in ED setting?

Our second research question revolves around the implementation process for TP in the ED setting. We found only 1 such study, which was conducted in a rural region of Australia, where the TP service was the main psychiatric service available [44]. The study was organized around the 6 concepts of the theory of access [46]. The following are the key findings, organized by these 6 concepts: (1) *Accessibility*: the staff were able to access mental health specialists for immediate assistance without transferring patients to another facility; (2) *Availability*: the service was valued as an available resource and eased the demands placed upon staff during emergency mental health presentations; (3) *Acceptability*: the service was acceptable to the providers and was a constant and easy resource; (4) *Affordability*: there were no direct costs borne by the providers or the hospitals to use TP to involve a psychiatrist, and it was free for the patients; (5) *Adequacy*: the 24-hour structure of the program was adequate to the clinical needs, particularly after-hours and on weekends; (6) *Awareness*: other than 1 provider, everyone else was aware of the service and had some experience using it [44]. In addition, the service provided a sense of security to the providers. They reported that before the service started, they had felt alone, unsupported, and lacking confidence when dealing with emergency mental health presentations [44]. All these improved due to the arrival of TP.

## Discussion

### Principal Findings

We performed a scoping review to examine the literature regarding the use of TP for adult emergencies. We summarized the evidence regarding (1) administrative and clinical outcomes for patients; and (2) process outcomes of implementing the TP service. Although TP is a known method for psychiatric evaluation, treatment, and follow-up, we found only 11 studies over the past decade to evaluate its application to the ED setting. Ten of these studies evaluated administrative and clinical outcomes, and only 1 study evaluated the implementation process.

Our review included articles that evaluated TP use in various settings and contexts. TP was acceptable and feasible nationally in the United States [15,19,37,38] and in a study of 7 Australian provinces [44]. We also found that TP was used in urban areas [41,42,45] and rural areas [40,47]. In some cases, TP was used in EDs as the only psychiatric service available [41]. The 1 study we found about the implementation process reported that TP was accepted and mostly appreciated by the ED staff, especially due to the lack of psychiatric expertise in their setting [44]. TP was also used for different sorts of patients, including those with anxiety and mood disorders and those with suicide or self-harm [43,45]. The identified lack of evidence regarding the use of TP in EDs significantly impacts our analysis, underscoring a crucial area where further research is needed to draw comprehensive conclusions. This gap highlights the limitations in our current understanding and emphasizes the necessity for targeted studies to elucidate the efficacy and implementation of TP in ED settings. Despite the limited number of studies we found, this diversity of settings and uses somewhat strengthens the argument that TP is broadly applicable across different ED settings and different patient groups.

### Waiting Times

On the issue of ED waiting times, the existing evidence is mixed. Two studies showed that the ED waiting time from patients’ arrival until psychiatric evaluation was significantly lower for TP visits than for in-person visits [41,43]. However, 2 studies found that the total ED LOS was significantly longer for TP visits than for in-person visits [19,43]. Unfortunately, none of the included studies examined waiting times from the psychiatric evaluation until admission or discharge for TP compared with in-person visits, so this remains an unexamined issue. However, because TP is shortening waiting times for initial psychiatric assessment, this may contribute to putting the patient on a better path from the beginning [29]. This is supported by the main finding of this review that TP reduced admission rates [19,40,42,43]. In other words, perhaps the fast psychiatric evaluation by TP resulted in expert psychiatric input to the case sooner, which may partly explain the lower admission rate. Further studies will help clarify these points.

### Patients’ Characteristics

Another factor that remains relatively unexamined is whether TP is equally applicable to different sorts of patients. Most enrolled studies did not examine TP use through different patient characteristics, such as diagnosis groups or the need for direct observation. Patients who require direct observation usually have more severe presentations and thus a longer LOS [48]. Two studies did focus on the use of TP for patients seen for self-harm and suicide; these studies showed higher ED LOS [19,43]. Therefore, there is a need for further studies of patients with these more severe presentations to ensure that TP is applicable to them as well.

### Lack of Findings Regarding Patients Evaluated for Involuntary Commitment

Patients requiring evaluation for involuntary commitment are a distinct group. As presented in the Results section, in 1 study, staff used TP to help evaluate which patients had improved enough to have their involuntary commitments converted into voluntary hospitalizations [39]. However, this does not speak to the initial decision to pursue an involuntary commitment. Given our group’s experience, it may be easy to understand why relatively few studies have evaluated the use of TP for patients evaluated for involuntary commitment. Our ongoing study of this issue required special permission from the Israeli Ministry of Health after consultation with the Ministry of Justice and the Union of Psychiatrists [49]. Thus, it is easy to see why there have been relatively few studies regarding the use of TP for this special use case and certainly more are needed.

### Patient Transfer

Another issue that was examined was the impact on interhospital transfers. One study found that the use of TP increased the number of transfers [43]. On the other hand, another study showed that among patients with an extended LOS, significantly less TP patients (29%) were transferred to a psychiatric hospital compared with in-person patients [40]. These divergent results may point to a complex and nuanced effect of TP on doctor-patient relationship. Technology in medicine holds the promise to contribute a more personalized style of care [50]. However, remote communication between psychiatrists and patients may affect doctor-patient engagement and lack personal touch compared with in-person encounters [51]. There is a possibility that it is easier for the psychiatrist using TP in emergency cases to decide on transfer rather than admission to the present facility. If there is an association between TP use in the ED and more patient transfers, this could lead to inconvenience for family members, as well as the cost of transport [52]. The impact of TP on the rate of interfacility transfers also requires further study.

### Rural and Urban Areas

TP is perceived often as a critical solution for the lack of mental health services in rural and remote areas [31,53]. However, findings from this review indicate that TP is used in urban areas as well [41,42]. Several studies showed that TP was even more common at urban settings [15,19], even when they have existing on-site psychiatric services [15]. The demand to use TP even in urban areas may be driven by the fact that the attending physician is at home for more hours than not, and must drive to the hospital. However, rural areas may face special issues with TP use, including inadequate technology literacy [2], bad internet connectivity [54], or a general lack of resources [2,55]. Despite these challenges, there is a strong incentive to use TP in rural areas, so it may be worth the effort of addressing the challenges.

### Strengths and Limitations

This scoping review had several strengths and limitations. A broad range of the main databases were searched, which allowed a comprehensive search. This review provides robust evidence of the included studies, provides a deeper understanding of the current evidence, and provides the needed data to broaden our understanding of TP in emergency settings.

This review also has some limitations. We examined only those studies published in English. All studies that we found were conducted in developed countries, which provides a limited perspective. In addition, the data we found about the use of TP for evaluations regarding possible involuntary commitment were particularly limited. This will be a key area for future research. We also did not find any studies that specifically compared TP use in urban versus rural settings, or that compared its use for specific groups of patients (eg, by diagnosis). Furthermore, we found only 1 interventional study; the others were observational. However, all our included studies had sample sizes larger than 30 participants and a detailed description of the study methodology. In part, we chose to do a scoping review as opposed to a systematic review, because the available literature was so limited.

### Conclusions

TP has a strong evidence base for general use and is known to be acceptable, reliable, and effective. However, only a very few studies in the past decade (11 studies) evaluated its use in the ED. While these studies generally supported the idea that TP was feasible and highly acceptable, it is clear that further studies are needed. Further studies are needed for examining TP evaluations for involuntary commitments in the ED setting. In addition, there is a need for studies on the extent and trends of TP usage over time, including in the context of COVID-19. We also need more comprehensive assessments comparing the effectiveness of TP evaluations with in-person assessments and implementation science research to better understand the barriers, facilitators, and opportunities for adopting this practice in EDs. Special attention should be given to rural areas, which usually have limited access to mental health services and yet may face special challenges in implementing them.

## Appendix

Multimedia Appendix 1 PRISMA (Preferred Reporting Items for Systematic Reviews and Meta-Analyses) checklist.

Multimedia Appendix 2 Web-based search strategy conducted on April 24, 2023.

## Abbreviations

ED: emergency department
LOS: length of stay
PRISMA: Preferred Reporting Items for Systematic Reviews and Meta-Analyses
PRISMA-ScR: Preferred Reporting Items for Systematic Reviews and Meta-Analyses extension for Scoping Reviews
TP: telepsychiatry
