# Supplementary material for: The Use of Telepsychiatry Services in Emergency Settings: Scoping Review
Source: J Med Internet Res. 2024 Jul 15;26:e51814. doi: 10.2196/51814 (PMC11287096; doi:10.2196/51814)
Supplement: Multimedia Appendix 1 [file jmir_v26i1e51814_app1.pdf]

Multimedia Appendix 1. PRISMA Extension for Scoping Reviews (PRISMA-ScR): Checklist and Explanation

SECTION	ITEM	PRISMA-ScR CHECKLIST ITEM	REPORTED ON PAGE #
TITLE		.	
Title	1	Identify the report as a scoping review.	page 1
ABSTRACT			
Structured summary	2	Provide a structured summary that includes (as applicable): background, objectives, eligibility criteria, sources of evidence, charting methods, results, and conclusions that relate to the review questions and objectives.	pages 1-2
INTRODUCTION			
Rationale	3	Describe the rationale for the review in the context of what is already known. Explain why the review questions/objectives lend themselves to a scoping review approach.	pages 4-6
Objectives	4	Provide an explicit statement of the questions and objectives being addressed with reference to their key elements (e.g., population or participants, concepts, and context) or other relevant key elements used to conceptualize the review questions and/or objectives.	page 6
METHODS			
Protocol and registration	5	Indicate whether a review protocol exists; state if and where it can be accessed (e.g., a Web address); and if available, provide registration information, including the registration number	No protocol
Eligibility criteria	6	Specify characteristics of the sources of evidence used as eligibility criteria (e.g., years considered, language, and publication status), and provide a rationale.	page 7 and Table 1
Information sources	7	Describe all information sources in the search (e.g., databases with dates of coverage and contact with authors to identify additional sources), as well as the date the most recent search was executed.	page 7
Search	8	Present the full electronic search strategy for at least 1 database, including any limits used, such that it could be repeated.	page 7; search strings can be found in Appendix 2
Selection of sources of evidence	9	State the process for selecting sources of evidence (i.e., screening and eligibility) included in the scoping review.	page 9; Figure 1
Data charting process	10	Describe the methods of charting data from the included sources of evidence (e.g., calibrated forms or forms that have been tested by the team before their use, and whether data charting was done independently or in duplicate) and any processes for obtaining and confirming data from investigators.	page 10
Data items	11	List and define all variables for which data were sought and any assumptions and simplifications made.	page 10
Critical appraisal of individual sources of evidence	12	If done, provide a rationale for conducting a critical appraisal of included sources of evidence; describe the methods used and how this information was used in any data synthesis (if appropriate).	Not applicable
Synthesis of results	13	Describe the methods of handling and summarizing the data that were charted.	page 10
RESULTS			
Selection of sources of evidence	14	Give numbers of sources of evidence screened, assessed for eligibility, and included in the review, with reasons for exclusions at each stage, ideally using a flow diagram.	page 10 to 11; Figure 1
Characteristics of sources of evidence	15	For each source of evidence, present characteristics for which data were charted and provide the citations.	page 11 to 19; Table 2

SECTION	ITEM	PRISMA-ScR CHECKLIST ITEM	REPORTED ON PAGE #
Critical appraisal within sources of evidence	16	If done, present data on critical appraisal of included sources of evidence (see item 12).	Not applicable
Results of individual sources of evidence	17	For each included source of evidence, present the relevant data that were charted that relate to the review questions and objectives.	pages 11 to 19
Synthesis of results	18	Summarize or present the charting results as they relate to the review questions and objectives.	pages 11 to 19
Summary of evidence	19	Summarize the main results (including an overview of concepts, themes, and types of evidence available), link to the review questions and objectives, and consider the relevance to key groups.	pages 19 to 24
Limitations	20	Discuss the limitations of the scoping review process.	page 23
Conclusions	21	Provide a general interpretation of the results with respect to the review questions and objectives, as well as potential implications or next steps.	page 24
FUNDING			
Funding	22	Describe sources of funding for the included sources of evidence, as well as sources of funding for the scoping review. Describe the role of the funders of the scoping review.	None
