# Supplementary material for: The Use of Telepsychiatry Services in Emergency Settings: Scoping Review
Source: J Med Internet Res. 2024 Jul 15;26:e51814. doi: 10.2196/51814 (PMC11287096; doi:10.2196/51814)
Supplement: Multimedia Appendix 2 [file jmir_v26i1e51814_app2.docx]

PubMed online search strategy conducted on 24 April 2023

| # | keywords |
| --- | --- |
| 1 | psychiat*[Title/Abstract] OR mental[Title/Abstract] |
| 2 | (("Psychiatry"[Mesh]) OR "Mental Health"[Mesh]) OR "Mental Disorders"[Mesh] |
| 3 | #1 OR #2 |
| 4 | telemedicine[Title/Abstract] OR "tele medicine"[Title/Abstract] OR "virtual medicine"[Title/Abstract] OR mhealth[Title/Abstract] OR telehealth[Title/Abstract] OR "tele health"[Title/Abstract] OR ehealth[Title/Abstract] OR telecare[Title/Abstract] OR "tele care"[Title/Abstract] OR "tele emergency"[Title/Abstract] OR "telemergency"[Title/Abstract] |
| 5 | "Telemedicine"[Mesh] |
| 6 | #4 OR #5 |
| 7 | #3 AND #6 |
| 8 | "tele psychiatry"[Title/Abstract] OR telepsychiatry[Title/Abstract] OR "tele mental"[Title/Abstract] OR telemental[Title/Abstract] OR "digital mental health"[Title/Abstract] |
| 9 | #7 OR #8 |
| 10 | Video*[Title/Abstract] OR "conference meeting"[Title/Abstract] OR streaming[Title/Abstract] OR zoom[Title/Abstract] OR "remote consultation"[Title/Abstract] OR "long distance consultation"[Title/Abstract] OR "distance counseling"[Title/Abstract] OR "e counseling"[Title/Abstract] OR "online counseling"[Title/Abstract] |
| 11 | "Videoconferencing"[Mesh] |
| 12 | #10 OR #11 |
| 13 | #9 AND #12 |
| 14 | (("Letter" [Publication Type]) OR "Editorial" [Publication Type]) OR "Comment" [Publication Type] |
| 15 | #13 NOT #14 |
| 16 | parent*[Title] OR paediatric*[Title] OR pediatric*[Title] OR child*[Title] OR adolescent*[Title] OR teen*[Title] OR youth[Title] OR juvenile[Title] OR school*[Title] |
| 17 | (group*[Title] OR couple*[Title] OR marital[Title] OR family[Title] OR offender*[Title] OR criminal*[Title] OR prisoner*[Title]) AND (support[Title] OR therapy[Title] OR counsel*[Title] OR psychotherapy[Title]) |
| 18 | "correctional setting"[Title/Abstract] OR "correctional facility"[Title/Abstract] OR jail[Title/Abstract] OR prison[Title/Abstract] OR "text based"[Title/Abstract] OR "text message"[Title/Abstract] |
| 19 | #16 OR #17 OR #18 |
| 20 | #15 NOT #19 |
| * | Filters: English, from 2013-2023 |

Web of Science online search strategy conducted on 24 April 2023

| # | keywords |
| --- | --- |
| 1 | psychiat* OR mental (Abstract) |
| 2 | psychiat* OR mental (Abstract) |
| 3 | #1 AND #2 |
| 4 | "tele psychiatry" OR telepsychiatry OR "tele mental" OR telemental OR "digital mental health" (Abstract) |
| 5 | #3 OR #4 |
| 6 | Video* OR "conference meeting" OR streaming OR zoom OR "remote consultation" OR "long distance consultation" OR "distance counseling" OR "e counseling" OR "online counseling" (Abstract) |
| 7 | #5 AND #6 |
| 8 | parent* OR paediatric* OR pediatric* OR child* OR adolescent* OR teen* OR youth OR juvenile OR school* (Title) |
| 9 | group* OR couple* OR marital OR family OR offender* OR criminal* OR prisoner* (Title) AND support OR therapy OR counsel* OR psychotherapy (Title) |
| 10 | "correctional setting" OR "correctional facility" OR jail OR prison OR "text based" OR "text message" (Abstract) |
| 11 | #8 OR #9 OR #10 |
| 12 | #7 NOT #11 |
| 13 | #7 NOT #11 and Article or Review Article (Document Types) |
| * | Filters: English, from 2013-2023 |

Embase online search strategy conducted on 24 April 2023

| # | keywords |
| --- | --- |
| 1 | psychiat*:ti,ab,kw OR mental*:ti,ab,kw |
| 2 | 'psychiatry'/exp OR 'mental disease'/exp OR 'mental health'/exp OR 'mental health care'/exp |
| 3 | #1 OR #2 |
| 4 | telemedicine:ti,ab,kw OR 'tele medicine':ti,ab,kw OR 'virtual medicine':ti,ab,kw OR mhealth:ti,ab,kw OR telehealth:ti,ab,kw OR 'tele health':ti,ab,kw OR ehealth:ti,ab,kw OR telecare:ti,ab,kw OR 'tele care':ti,ab,kw OR 'tele emergency':ti,ab,kw OR 'telemergency':ti,ab,kw |
| 5 | #3 AND #4 |
| 6 | 'tele psychiatry':ti,ab,kw OR telepsychiatry:ti,ab,kw OR 'tele mental':ti,ab,kw OR telemental:ti,ab,kw OR 'digital mental health':ti,ab,kw |
| 7 | 'telepsychiatry'/exp OR 'telemental health'/exp |
| 8 | #6 OR #7 |
| 9 | #5 OR #8 |
| 10 | 'video*':ti,ab,kw OR 'conference meeting':ti,ab,kw OR streaming:ti,ab,kw OR zoom:ti,ab,kw OR 'remote consultation':ti,ab,kw OR 'long distance consultation':ti,ab,kw OR 'distance counseling':ti,ab,kw OR 'e counseling':ti,ab,kw OR 'online counseling':ti,ab,kw |
| 11 | 'videoconferencing'/exp OR 'videophone'/exp |
| 12 | #10 OR #11 |
| 13 | #9 AND #12 |
| 14 | #13 AND ('Article'/it OR 'Article in Press'/it OR 'Review'/it) |
| 15 | parent*:ti OR paediatric*:ti OR pediatric*:ti OR child*:ti OR adolescent*:ti OR teen*:ti OR youth:ti OR juvenile:ti OR school*:ti OR ((group*:ti OR couple*:ti OR marital:ti OR family:ti OR offender*:ti OR criminal*:ti OR prisoner*:ti) AND (support:ti OR therapy:ti OR counsel*:ti OR psychotherapy:ti)) OR 'correctional setting':ab,ti OR 'correctional facility':ab,ti OR jail:ab,ti OR prison:ab,ti OR 'text based':ab,ti OR 'text messag*':ab,ti |
| 16 | 'child psychiatry'/mj |
| 17 | #15 OR #16 |
| 18 | #14 NOT #17 |
| * | Filters: English, from 2013-2023 |
